# Supplementary material for: Content-rich biological network constructed by mining PubMed abstracts
Source: BMC Bioinformatics. 2004 Oct 8;5:147. doi: 10.1186/1471-2105-5-147 (PMC528731; doi:10.1186/1471-2105-5-147)
Supplement: Additional File 2 — The original results of the above study (non-essential files are deleted to keep the file size under the limit set by BMC bioinformatics). [file 1471-2105-5-147-S2.bz2 › chilibotAdditionalFile2/dip40/451ID10477718E10462/html/CCL7_CCR5.html]

 


 **CCL7** and **CCR5** 
  
Found 35 abstracts in PubMed, retrieved 35.  
 

 What does Google say? 
 PDF only 
| .edu only 

---

**Interactive relationship** (e.g. stimulation, inhibition, etc)

**Inhibitory relationship**- In a parallel manner, binding of labeled MIP 1alpha was first shown to be attenuated by unlabeled RANTES, both of which recognize CCR1 and  **CCR5** , and then separately antagonized by MCP 3  [ **CCL7** ]  and MIP 1beta, which bind to CCR1 and  **CCR5** , respectively.  Ref: 10559781 Glia, 1999
- Among these ligands, MCP 3  [ **CCL7** ]  had the remarkable property of binding  **CCR5**  with high affinity without eliciting a functional response, MCP 3  [ **CCL7** ]  could also inhibit the activation of  **CCR5**  by MIP 1beta and may therefore be considered as a natural antagonist for  **CCR5** .  Ref: 10477718 Blood, 1999
**Neutral relationship**- Labeled MIP 1alpha binding was also antagonized by unlabeled MCP 3  [ **CCL7** ] , which is also recognized by CCR1, and MIP 1beta, which is a ligand for  **CCR5** .  Ref: 11032879 J Neurochem, 2000
- and c a MIP 1 alpha receptor  [ **CCR5** ]  that is activated by MIP 1 alpha, RANTES and, more weakly, by MCP 3  [ **CCL7** ] .  Ref: 7507512 J Exp Med, 1994
- **CCR5**  is most closely related to CCR2b, another chemokine receptor that responds to monocyte chemoattractant protein MCP 1, MCP 2, MCP 3  [ **CCL7** ] , and MCP 4.  Ref: 9312096 J Biol Chem, 1997

**Non-interactive relationship** (e.g. studied together, co-existance, homology, etc.)

- In parallel with down regulated SP and  **CCR5**  mRNA expression, cDNA array assays indicated that CP 9 45 treatment also inhibited IL 8 gene expression, while enhancing expression of fractalkine and monocyte chemotactic protein 3  [ **CCL7** ]  MCP 3  [ **CCL7** ] .  Ref: 12098517 J Neuroimmunol, 2002
- Recently we reported that the chemokines MIP 1alpha, MIP 1beta, and RANTES secreted by BM derived CD34 cells may compete with the macrophagotropic HIV R5 HIV strain for the  **CCR5**  coreceptor.In this study we extended our previous observations and examined various lympho hematopoietic CD34 cells isolated from thymus Th, cord blood CB, mobilized peripheral blood mPB, and BM for the expression of beta chemokines binding to  **CCR5** , i.e., MIP 1alpha, MIP 1beta, RANTES, MCP 2, MCP 3  [ **CCL7** ] , and MCP 4, and the alpha chemokine SDF 1 binding to CXCR4 as these chemokines may compete with the R5 and X4 HIV strains, respectively, for entry into cells.We found that Th, CB, mPB, and BM derived CD34 cells express mRNA transcripts for all the beta chemokines tested but not for SDF 1.  Ref: 11146155 Exp Hematol, 2000
- We investigated the effects of a number of naturally occurring chemokines MIP 1alpha, MIP 1beta, RANTES, MCP 2, MCP 3  [ **CCL7** ] , MCP 4 on different processes linked to the chemokine receptor  **CCR5**  in recombinant CHO cells expressing the receptor at different levels.  Ref: 11861332 Br J Pharmacol, 2002
- A number of inflammatory CC chemokines, including MIP 1 alpha, MIP 1 beta, RANTES, MCP 2, and HCC 1 9 74 act as  **CCR5**  agonists, while MCP 3  [ **CCL7** ]  is a natural antagonist of the receptor.  Ref: 12402506 Receptors Channels, 2002
- Similarly, the CCR2b ligands MCP 1 and MCP 3  [ **CCL7** ]  inhibit productive infection of PBMCs by both  **CCR5**  and CXCR4 dependent strains of HIV 1, suggesting that expression of the MCP 1 chemokine may affect HIV infection via signaling through the CCR2 receptor and subsequent desensitization of the  **CCR5**  and or CXCR4 signaling pathway.  Ref: 10497106 Virology, 1999
- also a ligand for  **CCR5** , or monocyte chemoattractant protein 3  [ **CCL7** ]  a ligand for CCR1 and CCR2b, nor was it able to induce Ca2 fluxes by itself.  Ref: 9334378 J Exp Med, 1997
- **CCR5**  binds multiple CC chemokines MCP 3  [ **CCL7** ]  acts as a natural antagonist.  Ref: 10477718 Blood, 1999
- Immunohistochemistry for MIP 1beta, MIP 1alpha, RANTES, eotaxin, and MCP 3  [ **CCL7** ]  ligands for  **CCR5**  and or CCR3 revealed the presence of MIP 1beta predominantly in a subpopulation of reactive astrocytes, which were more widespread in AD than control brains, and MIP 1alpha predominantly in neurons and weakly in some microglia in both AD and controls.  Ref: 9665462 Am J Pathol, 1998
- At 26 weeks postirradiation, messages encoding the chemokines BLC now known as Scyb13, C10 now known as Scya6  [ **CCL7** ] , IP 10 now known as Scyb10, MCP 1 now known as Scya2, MCP 3  [ **CCL7** ]  now known as Scya7  [ **CCL7** ] , MIP 1gamma now known as Scya9, and RANTES now known as Scya5 and the chemokine receptors Ccr1, Ccr2,  **Ccr5**  and Ccr6 were elevated in fibrosis sensitive C57BL 6 mice.  Ref: 11839087 Radiat Res, 2002
- MIP 1alpha, MIP 1beta, RANTES, MCP 2 and MCP 4 were able to stimulate increases in intracellular calcium ions via activation of  **CCR5**  whereas MCP 3  [ **CCL7** ]  was without effect.  Ref: 11861332 Br J Pharmacol, 2002
- In order to evaluate if these cells are infectabile by human immunodeficiency virus HIV and could be employed as a model in HIV research we exposed these cell lines to X4 T cell tropic and R5 macrophage tropic and subsequently tried to correlate their infectability with i level of chemokine coreceptor CXCR4 and  **CCR5**  expression, ii coreceptor functionality calcium flux, chemotaxis and phosphorylation of MAPK p42 44 and AKT and iii endogenous expression and secretion of HIV related chemokines which compete with the virus for binding to CXCR4 SDF 1 CXCL12 or  **CCR5**  MIP 1beta CCL4, MIP 1alpha CCL3, RANTES CCL5, MCP 2 CCL8, MCP 3  [ **CCL7** ]   **CCL7**  and MCP 4 CCL13.  Ref: 11737246 Eur J Haematol, 2001
